# Transmission of SARS-CoV-2 on cold-chain food overpacks: A new challenge

**DOI:** 10.7189/jogh.11.03071

**Published:** 2021-05-01

**Authors:** Yuhua Chi, Shiliang Zheng, Caide Liu, Qingxiu Wang

**Affiliations:** 1 Department of General Medicine, Affiliated Hospital of Weifang Medical University, Weifang, Shandong, China; 2 Department of Infection Management, Affiliated Hospital of Weifang Medical University, Weifang, Shandong, China

SARS-COV-2 has strong infectivity, fast transmission speed and peculiar transmission route. COVID-19 broke out all over the world in a very short time [1,2]. It was reported in Wuhan, China, in December 2019, mainly spread rapidly from person to person, and interpersonal communication mainly occurred in families [3]. This shows that close contact communication between individuals is one of the most important ways of transmission [4]. China has adopted strict quarantine measures and basically controlled the epidemic within a few months. Since July 2020, SARS-COV-2 has been detected frequently in imported cold chain food in China [5,6]. The spread of SARS-COV-2 carried by imported cold chain food immediately aroused widespread concern [7]. This paper analyzes and discusses the long-term survival of the virus in cold chain food and its cross-border transmission. It reminds that SARS-COV-2 can be directly transmitted to people by cold chain food packaging, and relevant personnel should pay attention to self-protection and isolation to prevent infection and transmission.

## CROSS-BORDER TRANSMISSION OF SARS-COV-2 ON COLD CHAIN FOOD PACKAGING

Although China has largely controlled the epidemic of COVID-19 in May 2020, it still faces the challenge of preventing imported infections from abroad in view of the fact that the epidemic continues in many countries. Due to the strict screening and isolation measures for the entry personnel, the imported cases were treated in time, which almost did not cause the widespread spread. In the second half of 2020, the spread of virus through imported cold chain food packaging occurred (**Table 1**) [7-10].

**Table 1:** Spread of SARS-COV-2 on the surface of the outer packaging of cold chain food imported from China

Time	City	Types of cold chain food	Number infected via cold chain	Number infected by cases infected via cold chain	Reference
3 Jul 2020	Qingdao, China	frozen cod	2	12 people were infected in hospital	[7]
9 Nov 2020	Tianjin, China	N/A	2	No community transmission has occurred	[8]
17 Dec 2020	Dalian, China	frozen cod	2	No community transmission has occurred	[9]
22 Jul 2020	Dalian, China	A variety of seafood	3	79 case of regional infections (from Mr. Shi's workplace and the surrounding communities of his company)	[10]

In severe COVID-19 areas, patients with latent period and asymptomatic infections may cause contamination in the processing, packaging, handling and transportation of cold chain food, resulting in the existence of SARS-CoV-2 on frozen food and its surface. SARS-COV-2 on the surface of contaminated frozen food may remain active after long-distance transportation to China.

Since July 2020, SARS-COV-2 has been found, in Dalian, Qingdao and other cities in China, on the surface and in containers of cold chain food packages from South America and other regions [5,6]. From July to October 2020, Dalian Customs detected SARS-COV-2 from the outer packaging surface of frozen South American white shrimp from Ecuador. On July 22, Mr. Shi, who was engaged in the processing of imported and domestic seafood in Dalian Kaiyang World Seafood Co. Ltd, was infected. Two days later, two workers in the same workshop were also confirmed. Since then to August 5, 79 people have been infected, who are mainly from Mr. Shi’s workplace and the surrounding communities of his company [10]. Before that, SARS-COV-2 was not found in the outer packaging of imported cold chain food, and the relevant management system and measures were not perfect. Mr. Shi did not inspect and record the imported cold chain food and processed food packages he handled. Although it is suspected that the infection of Mr. Shi and others is related to the spread of imported cold chain food, there is no relevant evidence.

SARS-CoV-2 were isolated from the imported frozen cod outer package's surface, which showed that imported frozen food industry could import SARS-CoV-2. It indicated that cases in Qingdao were probably caused by SARS-CoV-2 contamination of cod outer package during production or cold-chain transportation [5,7].

On September 24, 2020, during the routine nucleic acid inspection of the personnel in Qingdao Port, two stevedores were found to be SARS-CoV-2 positive and were identified as asymptomatic infection. It was revealed that both cases had no COVID-19 case contact history and no foreign personnel contact history. However, both carried out loading and unloading of frozen cod in bulk on September 19, 2020.

Subsequently, the surface swab samples of the frozen cod outer package were collected and then tested. Out of 421 surface samples, 50 were tested SARS-CoV-2 nucleic acid positive. After that, the whole-genome sequencing and virus isolation were performed on the throat swab samples taken from the two workers and the frozen cod outer package's surface swab samples. Phylogenetic analysis indicated that the SARS-CoV-2 resulted in the outbreak in Qingdao City fell in a European branch (L lineage B1.1), which originated in Europe. The virus' full genome sequence isolated from the patient's nasopharyngeal swab has a 100% similarity to that of the original sample. In contrast, the virus' genome sequence is highly homologous to the original piece from the frozen cod outer package, with two distinct nucleotides [5,7].

One of the two asymptomatic patients who came into contact with imported cold chain food developed fever, cough and other clinical symptoms during hospital isolation, and was diagnosed as COVID-19 later. He underwent a computerized tomography (CT) examination without standard disinfection and protection measures. Two tuberculosis patients went for CT examination the next day and were infected. After that, they infected 10 people in the ward [7].

Since then, in Tianjin city and Dalian city of China, it has been found that the staff were infected with COVID-19, who had handled the outer packaging of cold chain food with positive SARS-COV-2 [8,9].

## DISSCUSSION

Unlike other viruses, SARS-COV-2 can survive for a period of time after leaving the host. In addition to its own characteristics, the survival time of the virus away from the host mainly depends on the physical and chemical properties of the surface and the environmental conditions (mainly climate, light, temperature, humidity and so on.) [11-13]. Chin and other scholars [12] reported that SARS-COV-2 survived for 7 days on the plastic surface, 4 days on the stainless steel surface at room temperature, and relatively short on commonly used paper documents, banknotes and mail wrapping paper.

**Figure:**
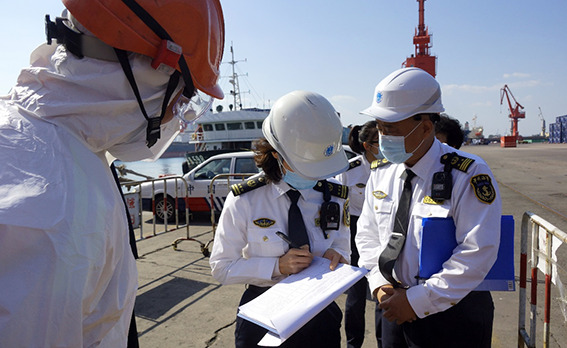
Photo: SARS-COV-2 can be spread across the border through cold chain food for a long distance. It is necessary to strictly check and record the imported cold chain food (from the collection of Denglao Wangdonghai, used with permission).

The longest survival time of SARS-COV-2 has not been determined under specific conditions such as cold chain products and other low temperatures [12]. When SARS-COV-2 was implanted into salmon, chicken and pork slices bought in supermarkets, the number of virus remained unchanged at -20°C for three weeks [14]. In view of the fact that cold chain food is in the environment below -18°C, SARS-COV-2 can survive on its outer packaging surface for a long time. After contacting the cold chain food and its outer packaging surface, unprotected personnel may pick their nose, rub their eyes and touch their lips, thus infecting COVID-19. In Dalian, China, a person who processed imported cold chain food was infected, and 79 people were infected one after another [10]. In Qingdao, 12 people were infected by the person who contacted with imported cold chain food, which indicates that the person who is engaged in importing cold chain food cannot only be infected with COVID-19, but also cause the spread of the virus [7]. In order to prevent the spread of COVID-19 via imported cold chain food packaging, it is necessary to strictly implement the national policy of SARS-COV-2 cold chain food prevention and treatment, wear work clothes, work caps, disposable medical masks, gloves and avoid goods close to the face and hands touching the nose and mouth. Before leaving the cold chain food workplace, it is inevitable to disinfect, follow the health registration system and abnormal health reporting procedures of imported cold chain food practitioners, and conduct nucleic acid detection once a week. The imported cold chain processors from Qingdao and Tianjin were identified in routine nucleic acid screening, which also shows the importance of comprehensive nucleic acid detection in the processing of imported cold chain food.

Although respiratory droplets, air transmission and direct contact are the main modes of transmission, there are animal to human and human to animal transmission [15]. The main route of transmission is person to person. There are few reports about animals infecting humans, and there are reports about minks infecting humans [16]. It is also rare for goods to reach other places and then infect people [17].

SARS-COV-2 on the outer packaging surface of cold chain food can exist for a long time and spread across the border for a long distance. Although there is a lack of systematic research, and these cases are rare, it still reminds people to guard against the virus spread of cold chain food [18].

## CONCLUSION

SARS-COV-2 can survive on cold chain food and its packaging surface for a long time, which may lead to long-distance transmission. Therefore, we should be highly alert to the cross-border spread of COVID-19 caused by the import of cold chain food.
